# Bronchiectasis Is a Model for Chronic Bacterial Infection Inducing Autoimmunity in Rheumatoid Arthritis

**DOI:** 10.1002/art.39226

**Published:** 2015-08-26

**Authors:** Anne‐Marie Quirke, Elizabeth Perry, Alison Cartwright, Clive Kelly, Anthony De Soyza, Paul Eggleton, David Hutchinson, Patrick J. Venables

**Affiliations:** ^1^ Kennedy Institute, Nuffield Department of Orthopaedics, Rheumatology and Musculoskeletal Sciences, University of OxfordOxfordUK; ^2^ Queen Elizabeth Hospital, Gateshead, UK, and University of Exeter Medical SchoolExeterUK; ^3^ Queen Elizabeth HospitalGatesheadUK; ^4^ Newcastle University and The Freeman HospitalNewcastleUK; ^5^ University of Exeter Medical SchoolExeterUK; ^6^ Royal Cornwall HospitalTruroUK

## Abstract

**Objective:**

To examine the potential of chronic severe bacterial infection to generate rheumatoid factor (RF) and anti–citrullinated protein antibodies (ACPAs), by studying patients with bronchiectasis (BR) alone and BR patients with rheumatoid arthritis (BR/RA).

**Methods:**

We studied 122 patients with BR alone, 50 patients with BR/RA, and 50 RA patients without lung disease, as well as 87 patients with asthma and 79 healthy subjects as controls. RF levels were measured using an automated analyzer, and cyclic citrullinated peptide 2 (CCP‐2) was used to detect ACPAs. The fine specificities of citrullinated α‐enolase peptide 1 (CEP‐1), Cit‐vimentin, and Cit‐fibrinogen to their arginine‐containing control peptides (arginine‐containing α‐enolase peptide 1 [REP‐1], vimentin, and fibrinogen) were measured by enzyme‐linked immunosorbent assay.

**Results:**

Among the BR patients and control subjects, 39% and 42%, respectively, were ever‐smokers. The frequency of RF positivity in serum was increased in BR patients compared with controls (25% versus 10%), as were the frequencies of antibodies to CCP‐2 (5% versus 0%), CEP‐1 (7% versus 4%), Cit‐vimentin (7% versus 4%), and Cit‐fibrinogen (12% versus 4%), although only the differences for RF and Cit‐fibrinogen were significant (*P* < 0.05). We observed a corresponding increase in the frequency of antibodies to the arginine‐containing control peptides in BR patients compared with controls (for REP‐1, 19% versus 4% [*P* < 0.01]; for vimentin, 16% versus 4% [*P* < 0.05]), demonstrating that the ACPA response in patients with BR is not citrulline specific. The lack of citrulline specificity was further confirmed by absorption studies. In BR/RA patients, all ACPA responses were highly citrulline specific.

**Conclusion:**

Bronchiectasis is an unusual but potent model for the induction of autoimmunity in RA by bacterial infection in the lung. Our study suggests that the ACPA response is not citrulline specific during the early stages of tolerance breakdown but becomes more specific in patients with BR in whom BR/RA develops.

Rheumatoid arthritis (RA) is an autoimmune disease characterized by the presence of disease‐specific anti–citrullinated protein antibodies (ACPAs) 1. Because ACPAs can be detected in patients with RA several years before the diagnosis is made 2, it is now thought that RA‐related autoimmunity may be initiated outside the joint, in sites such as the lungs and the periodontium 3, 4.

Smoking is a known risk factor for RA 3, 5. There is accumulating evidence that the ACPA response results from smoking‐induced inflammation of the lung, resulting in increased expression of citrullinated proteins 6, 7. Periodontitis, which often is cited as one of the most common inflammatory diseases, is also a risk factor for RA 8, and patients with periodontitis have increased levels of antibodies against the uncitrullinated forms of RA autoantigens 9, 10.

Bronchiectasis (BR) has been recognized as a risk factor for RA since publication of the classic studies by Walker nearly 50 years ago 11. He observed that among 516 patients with RA, 2.5% had symptoms of antecedent BR compared with 0.3% of 300 patients with degenerative joint disease. Similar findings have been observed in other cohorts of patients with RA 12. Importantly, in a more recent study, RA developed in 2 patients with BR over 12 months of followup 13. Although it would be difficult to confidently calculate the relative risk in these studies, it would be fair to conclude that BR is a potent risk factor for RA in a minority of patients. Similar to other severe chronic bacterial infections, BR has been known for decades to be associated with a high frequency of rheumatoid factors (RFs) 14, 15, suggesting that chronic bacterial infection of the lung could lead to autoimmunity in RA. However, there are no published studies of the fine specificity of ACPAs in BR, and the potential mechanisms of citrulline‐specific autoimmunity induced by bacterial infection have not been studied in BR.

In this study, we used BR as a model to study the evolution of the ACPA response induced by severe chronic bacterial infection, as 2 cross‐sectional “snapshots” at the beginning and the end of development of the ACPA response, in patients with BR and BR patients in whom RA later develops. To assess whether BR could be a model for the induction of autoimmunity in RA, we measured the levels of autoantibodies to both citrullinated and uncitrullinated peptides in a well‐documented group of BR patients without RA, using healthy subjects and patients with asthma as controls. To examine the ACPA response in patients with established disease, we measured the levels of these autoantibodies in BR patients with concomitant RA (BR/RA) and in RA patients without any lung disease.

## PATIENTS AND METHODS

### Serum samples from patients and control subjects

Serum samples from 122 patients with BR, 50 patients with BR/RA, 50 RA patients without lung disease, 87 patients with asthma, and 79 healthy control subjects were obtained from several centers across the UK 13. All of the patients with BR were adults (age >18 years) with high‐resolution computed tomography (HRCT)–proven symptomatic non–cystic fibrosis BR and a history of ≥2 respiratory infections per year. HRCT was performed by chest radiologists who were not involved in the study, and all patients in the BR cohorts were receiving followup care from a respiratory consultant (a physician with an interest in BR). Patients with coexistent intestinal lung disease, asthma, emphysema, allergic bronchopulmonary aspergillosis, a history of or current tuberculosis, or those in whom any other lung disease was observed by HRCT or reported in respiratory clinic notes (referencing electronic letters and patients’ paper notes taken at the respiratory clinic) were excluded from the study. All BR patients also underwent a musculoskeletal examination by a rheumatologist and were excluded if they had a history of inflammatory joint pain, inflammatory arthritis, or any synovitis 13. All RA patients met the American College of Rheumatology/European League Against Rheumatism 2010 criteria for RA 16. RF was present in significantly greater numbers of BR/RA patients compared with patients with RA alone 13. Multicenter ethics approval was obtained at all participating centers (Integrated Research Application System approval no. 12324). Recruitment was performed between May 2012 and May 2013.

### Antibody measurements

The levels of IgM RFs were measured with an Hitachi Modular P analyzer and were defined as positive or negative according to the cutoff levels recommended by the manufacturer. All of the ACPA tests were performed in the same laboratory by a single investigator (A‐MQ). Anti–cyclic citrullinated peptide 2 (anti–CCP‐2) antibody levels were measured using a commercial enzyme‐linked immunosorbent assay (ELISA) (Diastat; Eurodiagnostica) according to the instructions of the manufacturer. Antibodies to immunodominant peptides from 3 established RA autoantigens, citrullinated α‐enolase peptide 1 (CEP‐1; amino acids [aa] 4–21 [KIHA‐cit‐EIFDS‐cit‐GNPTVE]) 17, Cit‐vimentin (aa 59–74 [VYAT‐cit‐SSAV‐cit‐L‐cit‐SSVP]), and Cit‐fibrinogen β‐chain (aa 36–52 [NEEGFFSA‐cit‐GHRPLDKK]) 10 were also measured by in‐house ELISAs, as previously described 10. For all assays, arginine‐containing control peptides were run in parallel. For the immunodominant peptides and control peptides, we calculated the cutoff for positivity as the ninety‐fifth percentile of the value for normal controls, and all serum samples with values above this cutoff were considered positive. A standard curve was included in all plates coated with citrullinated peptides and those coated with uncitrullinated vimentin, and results were expressed as arbitrary units. For assays without standard curves, the results were expressed as the optical density (OD).

### Inhibition/competition assays

Inhibition/competition experiments were performed in the liquid phase. Six anti–arginine‐containing α‐enolase peptide 1 (anti–REP‐1)–positive serum samples from patients with BR and 6 anti–CEP‐1–positive samples from patients with BR/RA were chosen. Sera were incubated for 2 hours at room temperature and overnight at 4°C in buffer alone as control or in 1 mg/ml of either REP‐1 or CEP‐1. The mixtures were centrifuged at 16,200*g* for 20 minutes, the BR supernatants were transferred to REP‐1–coated plates and the BR/RA supernatants were transferred to CEP‐1–coated plates, and assays were performed as described above. All results were expressed as the OD at 450 nm.

### Statistical analysis

The Mann‐Whitney nonparametric test (for unmatched groups) was used to compare differences between antibody responses in the cohorts of serum samples. Spearman's nonparametric correlations between data sets were assessed. Calculations were performed using GraphPad software.

## RESULTS

### Demographic characteristics of the study participants

The demographic characteristics of the patients and control subjects are shown in Table 1. The patients were well matched for sex, with a preponderance of women in all groups, although the median age of subjects in the control group was ∼7 years younger than that of patients with BR, patients with BR/RA, and patients with RA. Ever‐smoking was reported by 39% of the BR patients without RA, 42% of the control subjects, 43% of the patients with asthma, and 56% of the patients with RA only. The frequency of RF was significantly increased in patients with BR (25%) compared with healthy controls (10%), as previously observed 14, 15. As expected, highly significant increases in RF positivity were observed in both the RA group and the BR/RA group compared with controls (Table 2).

**Table 1:** Demographic characteristics of the healthy control subjects and patients^a^

	n	Age, median (IQR) years	Female sex, %	Ever‐smoker, %
Healthy controls	79	60 (17)	74	42
Patients				
Asthma	87	50 (27)	79	43
BR	122	66 (13)	65	39
BR/RA	50	68 (12)	72	42
RA	50	66 (15)	72	56

a There were no significant differences in age between patients with bronchiectasis (BR), BR patients with rheumatoid arthritis (BR/RA), and patients with RA. Patients with asthma were significantly younger than subjects in all other groups. Healthy control subjects were significantly younger then patients in the BR/RA and RA groups. There were no significant differences in sex or smoking status between all groups, with the exception of the group with asthma, in which the percentage of female patients was significantly higher than that in the group with BR.

**Table 2:** Serum antibody positivity in the healthy control subjects and patients^a^

Antibody	Healthy controls (n = 79)	Patients			
		Asthma (n = 87)	BR (n = 122)	BR/RA (n = 50)	RA (n = 50)
Rheumatoid factor	10	16	25^b^	82^c^	52^c^
CCP‐2	0	1	5	88^c^	48^c^
CEP‐1	4	1	7	60^c^	24^d^
Cit‐vimentin	4	3	7	56^c^	20^d^
Cit‐fibrinogen	4	0	12^b^	74^c^	40^c^
REP‐1	4	3	19^d^	12	0
Vimentin	4	3	16^b^	20^d^	2
Fibrinogen	4	2	9	6	8

a Values are the percent. BR = bronchiectasis; BR/RA = BR with rheumatoid arthritis (RA); CCP‐2 = cyclic citrullinated peptide 2; CEP‐1 = citrullinated α‐enolase peptide 1; REP‐1 = arginine‐containing α‐enolase peptide 1.

b *P* < 0.05 versus controls, by Fisher's exact test.

c *P* < 0.001 versus controls, by Fisher's exact test.

d *P* < 0.01 versus controls, by Fisher's exact test.

### Non–citrulline‐specific ACPAs in BR patients without RA

**Figure 1:**
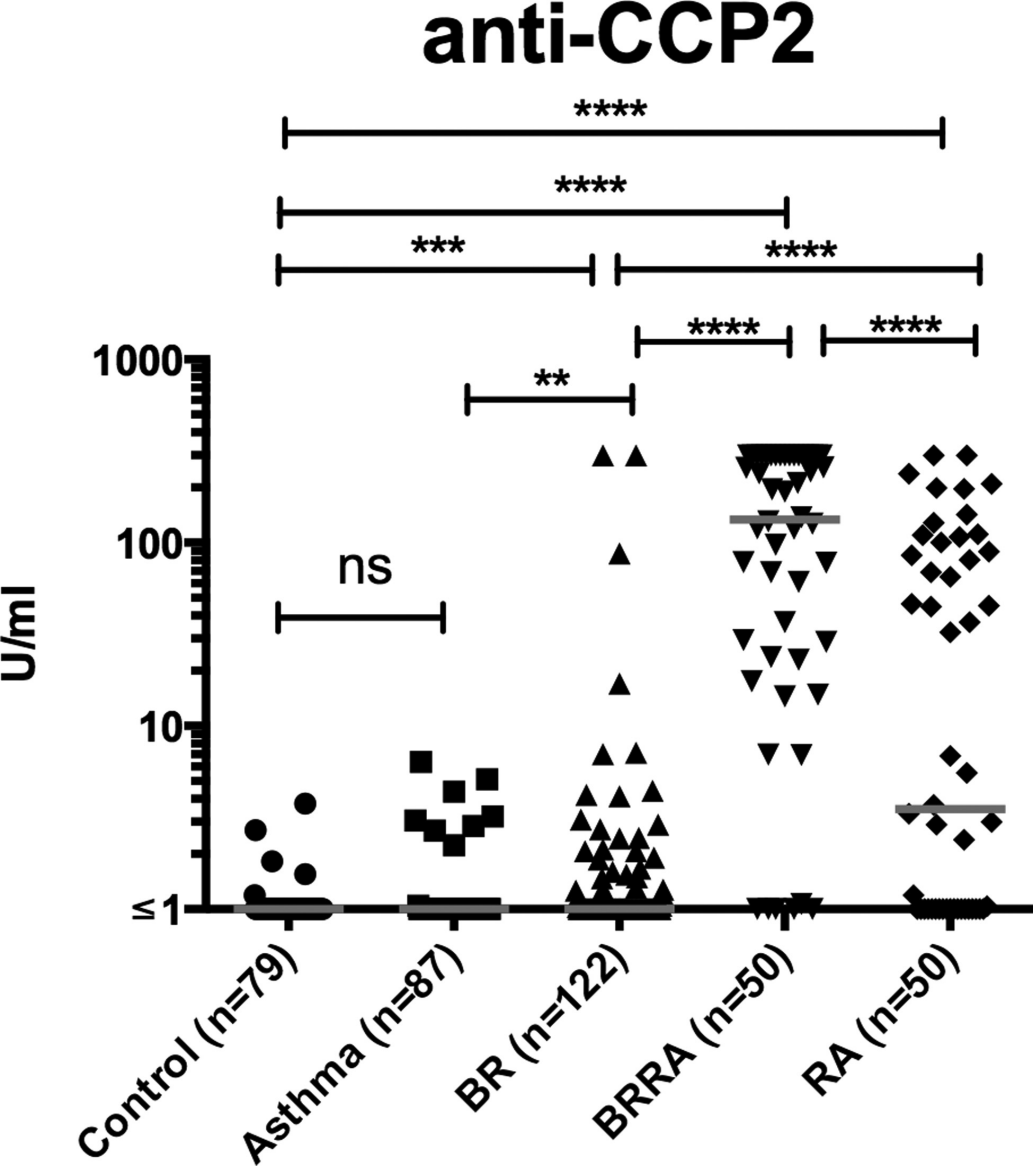
Anti**–**cyclic citrullinated peptide 2 (anti–CCP‐2) levels in healthy control subjects, patients with asthma, patients with bronchiectasis (BR), BR patients with rheumatoid arthritis (BR/RA), and patients with RA. Each symbol represents an individual subject; horizontal lines show the median. NS = not significant. ∗∗ = *P* < 0.01; ∗∗∗ = *P* < 0.001; ∗∗∗∗ = *P* < 0.0001.

**Figure 2:**
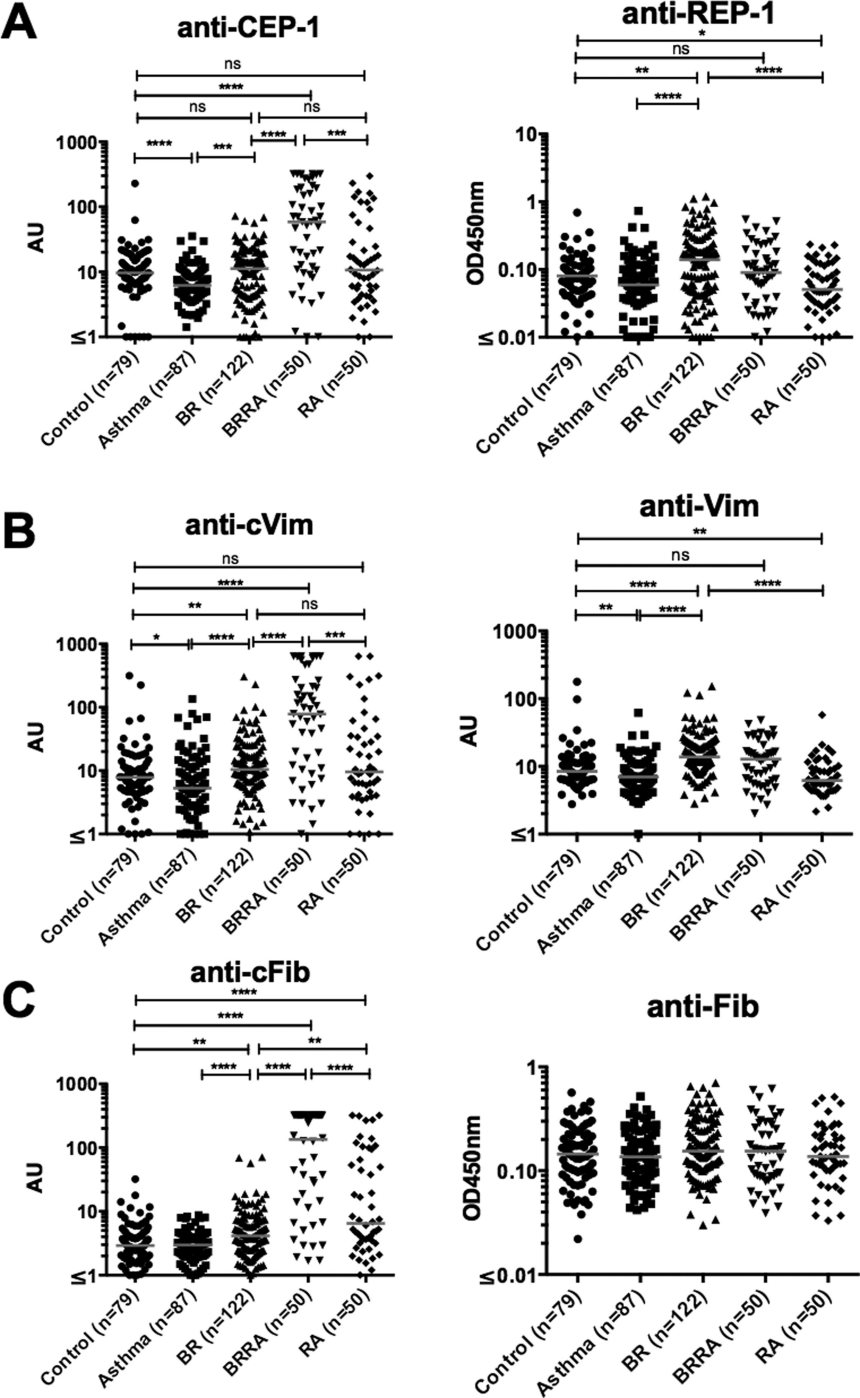
Levels of anti–citrullinated α‐enolase peptide 1 (anti–CEP‐1) and anti–arginine‐containing α‐enolase peptide 1 (anti–REP‐1) (**A**), anti–Cit‐vimentin (anti‐cVim) and anti‐vimentin (**B**), and anti–Cit‐fibrinogen (anti‐cFib) and anti‐fibrinogen (**C**) in healthy control subjects, patients with asthma, patients with BR, patients with BR/RA, and patients with RA. Each symbol represents an individual subject; horizontal lines show the median. ∗ = *P* < 0.05; ∗∗ = *P* < 0.01; ∗∗∗ = *P* < 0.001; ∗∗∗∗ = *P* < 0.0001. See Figure 1 for other definitions.

Six of the BR patients (5%) were anti–CCP‐2 antibody positive. Although the rates of anti–CCP‐2 positivity were not significantly different between BR patients and controls (Table 2), the titers of anti–CCP‐2 antibodies in BR patients were significantly increased compared with those in both healthy controls (*P* < 0.001) and patients with asthma (*P* < 0.01) (Figure 1). The frequencies of antibody positivity to the “specific” citrullinated peptides were slightly increased in patients with BR compared with controls (for CEP‐1, 7% versus 4%; for Cit‐vimentin, 7% versus 4%; for Cit‐fibrinogen, 12% versus 4%); only the difference in the frequency of antibodies to Cit‐fibrinogen reached statistical significance (*P* < 0.05) (Table 2). When comparing the titers of antibody binding, the levels of antibodies to all 3 peptides were significantly increased in BR patients compared with patients with asthma and, in the case of both anti‐vimentin and anti–Cit‐fibrinogen, compared with healthy controls (Figure 2).

**Figure 3:**
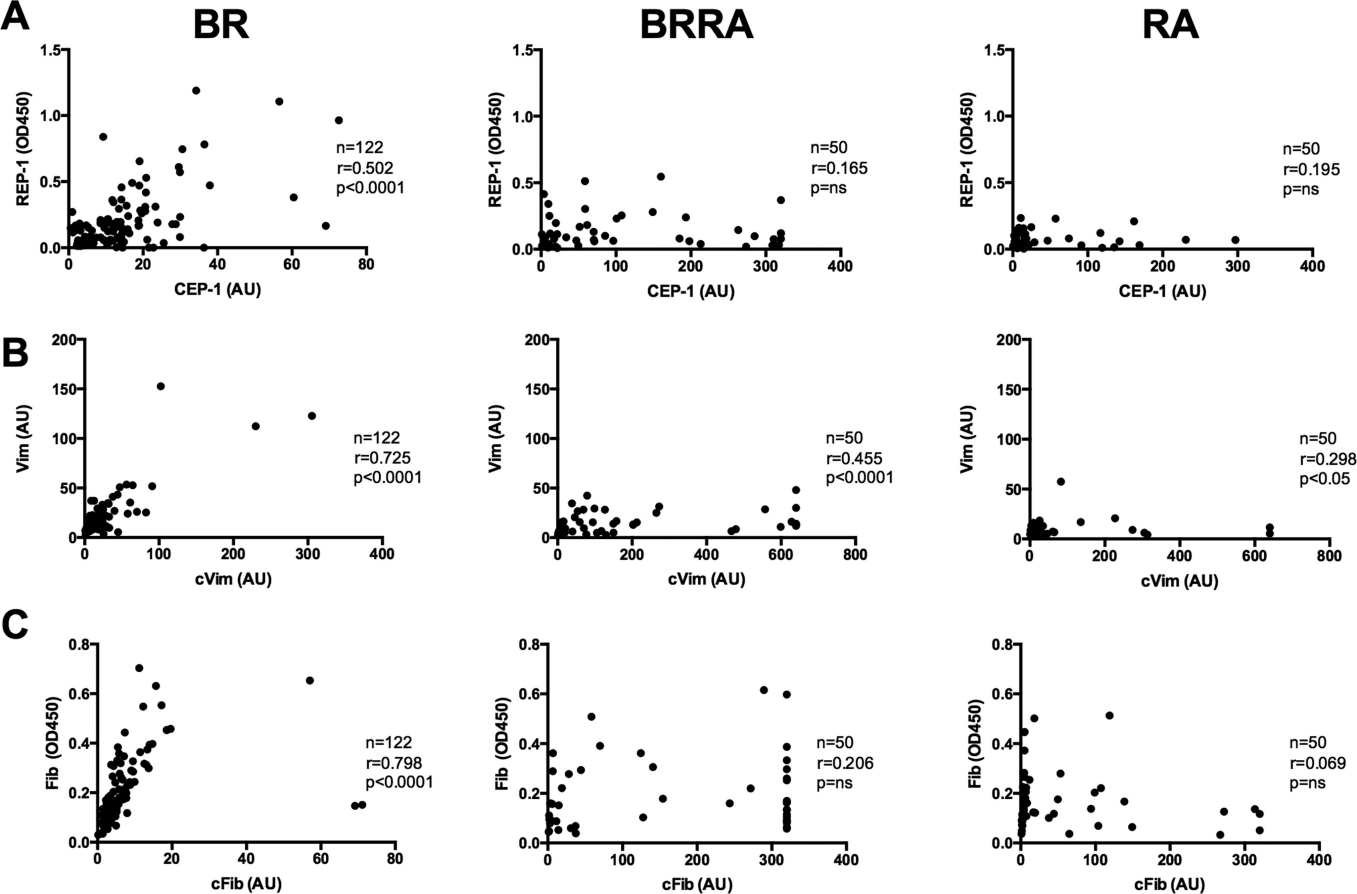
Correlations between the levels of antibodies to specific anti–citrullinated protein antibodies and their arginine‐containing control peptides in patients with BR, patients with BR/RA, and patients with RA. **A,** Anti–citrullinated α‐enolase peptide 1 (anti–CEP‐1) responses versus anti–arginine‐containing α‐enolase peptide 1 (anti–REP‐1) responses. **B,** Anti–Cit‐vimentin (anti‐cVim) responses versus anti‐vimentin responses. **C,** Anti–Cit‐fibrinogen (anti‐cFib) responses versus anti‐fibrinogen responses. See Figure 1 for other definitions.

The frequencies of antibodies to the arginine‐containing control peptides from the specific citrullinated antigens were also increased in patients with BR compared with healthy controls (for REP‐1, 19% versus 4%; for vimentin, 16% versus 4%; for fibrinogen, 9% versus 4%) (Table 2). In addition, the titers of anti–REP‐1 and anti‐vimentin were significantly increased in patients with BR compared with both healthy controls and patients with asthma (Figure 2). The lack of citrulline‐specific ACPA responses in the BR patients was further suggested by the strong correlations between antibodies to each of the citrullinated peptides compared with their uncitrullinated variants (for anti–CEP‐1 versus anti–REP‐1, r = 0.502 [*P* < 0.0001]; for anti–Cit‐vimentin versus anti–vimentin, r = 0.725 [*P* < 0.0001]; for anti–Cit‐fibrinogen versus anti‐fibrinogen, r = 0.798 [*P* < 0.0001]) (Figure 3).

**Figure 4:**
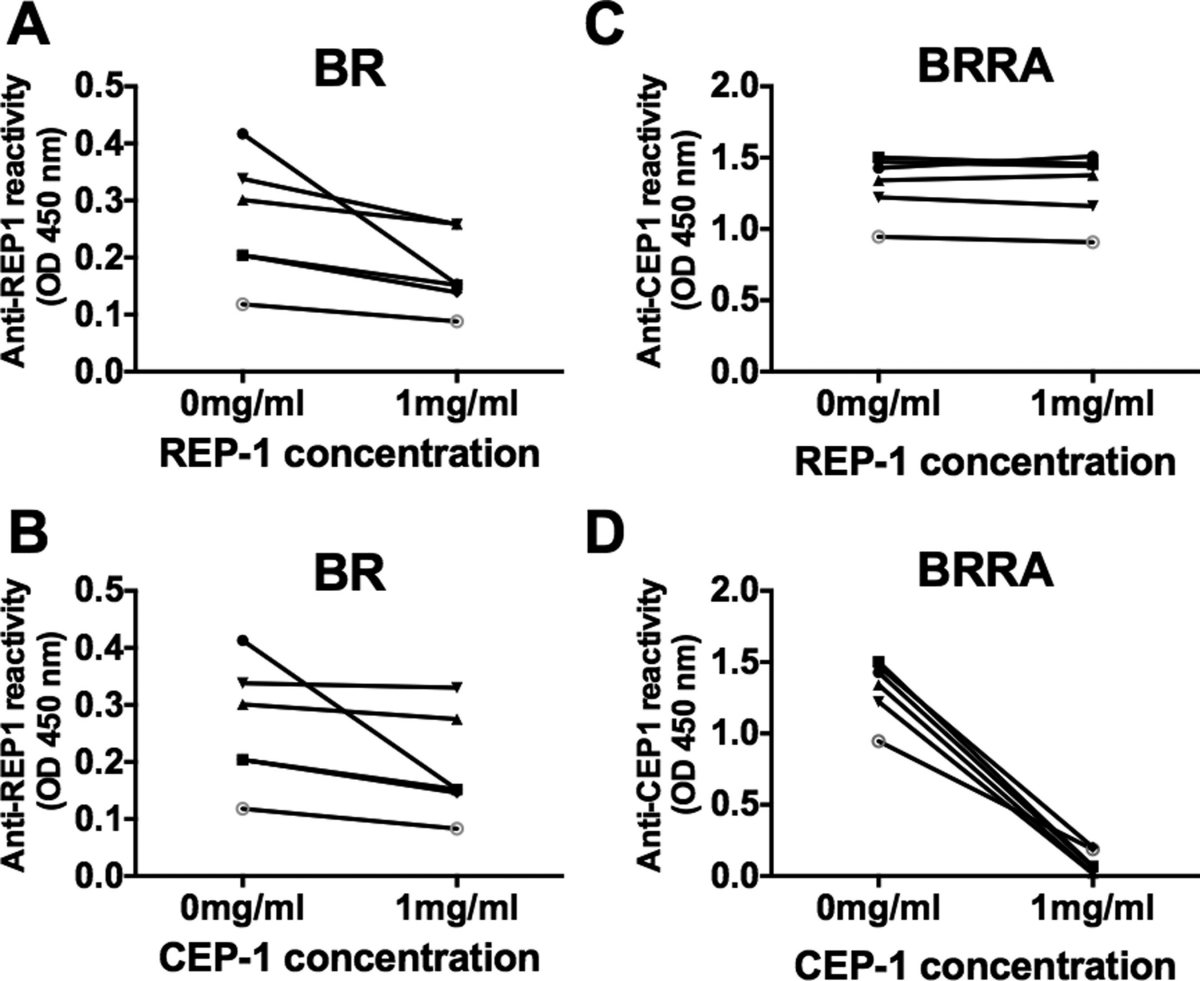
Antibody responses in anti–REP‐1–seropositive patients with bronchiectasis (BR) and in anti–CEP‐1–seropositive BR patients with rheumatoid arthritis (BR/RA) in the presence of either REP‐1 or CEP‐1, as determined by enzyme‐linked immunosorbent assay–based competition/inhibition assays. **A** and **B,** Anti–REP‐1 reactivity in the presence of REP‐1 and CEP‐1 in patients with BR. **C** and **D,** Anti–CEP‐1 reactivity in the presence of REP‐1 and CEP‐1 in patients with BR/RA. Each symbol represents a different patient; lines connect the individual data points. See Figure 2 for other definitions.

The citrulline specificity of the antibody response in patients with BR was also examined by performing absorption experiments with the 2 enolase peptides, CEP‐1 and REP‐1, in 6 of the serum samples from BR patients. The anti–REP‐1 response in these sera was absorbed partially and almost equally with both REP‐1 and CEP‐1 (Figures 4A and B), suggesting that antibodies were cross‐reactive for both peptides and further confirming their lack of citrulline specificity.

### Citrulline specificity and ACPA levels in patients with BR/RA

Compared with the patients with BR, those with BR/RA had a highly citrulline‐specific ACPA response to each antigen tested (Figures 1 and 2). In addition, no significant correlations between the antibody responses to the citrullinated peptides and the arginine‐containing control peptides were observed in patients with BR/RA, with the exception of the correlation between anti–Cit‐vimentin and anti‐vimentin (r = 0.455 [*P* < 0.0001]) (Figure 3).

Although the percentage of ever‐smokers was reduced among patients with BR/RA compared with that among patients with RA alone (42% versus 56%; *P* = 0.06), the rate of seropositivity in BR/RA patients compared with RA patients was significantly increased for each ACPA tested: for anti–CCP‐2, 88% versus 48%; for anti–CEP‐1, 60% versus 24%; for anti–Cit‐vimentin, 56% versus 20%; and for anti–Cit‐fibrinogen, 74% versus 40% (Table 2) (all *P* < 0.01). The citrulline specificity of the ACPA response in patients with RA was confirmed by the lack of correlation with the response to the arginine‐containing peptides (Figures 2 and 3) and by complete absorption of the anti–CEP‐1 response in 6 BR/RA serum specimens only with the CEP‐1 peptide (Figures 4C and D). Taken together, these data showed that the citrulline specificity of ACPAs in patients with BR/RA was increased compared with that in patients with BR alone, and its magnitude was increased compared with that in RA patients without any lung disease.

## DISCUSSION

Our study showed significantly elevated levels of ACPAs in patients with BR, although the antibody response was not citrulline specific. We suggest that after tolerance has been breached, subsequent long‐term exposure to citrullinated antigens in the inflamed lung results in the spreading of epitopes recognized by B cells to citrullinated peptides. Epitope spreading in patients with BR in whom RA develops may well be reflected by the markedly citrulline‐specific responses to CCP‐2, CEP‐1, Cit‐fibrinogen, and Cit‐vimentin observed in the BR/RA patients. We also observed an increased frequency of RF positivity in patients with BR, but this has been well described in previous studies 14, 15. Furthermore, RFs can also be found in patients with other autoimmune and nonautoimmune conditions, as well as in healthy individuals (for review, see ref. 18). The increased titers and frequencies of both ACPAs and RF in patients with BR/RA compared with patients with RA may also reflect the potency of BR in exacerbating the autoimmune response in RA, once it has developed.

Our data suggest that this effect could not be explained by cigarette smoking, which is a well‐established risk factor for RA. In our study the percentage of ever‐smokers was actually lower among BR patients compared with controls, although it was increased slightly among the RA patients without lung disease. However, these data must be interpreted with caution, because “ever smoking” is not regarded as a particularly robust measure of tobacco exposure 19.

The largely arginine‐specific autoantibody response that we observed in patients with BR was remarkably similar to that observed in patients with periodontitis 10. This pattern of response was also recently observed in a study by Brink et al 20, in which antibodies against arginine‐containing peptides (including REP‐1, vimentin [aa 60–75], and fibrinogen β‐chain [aa 36–52]) from RA autoantigens were observed before the development of citrulline‐specific ACPAs in serum samples obtained from patients with RA years before symptoms of RA developed. Importantly, that study also included sequential samples. However, it was entirely cross‐sectional, and further prospective investigations will need to be carried out in BR patients at risk of RA in order to confirm the evolution of the citrulline specificity of ACPAs in patients with BR in whom BR/RA subsequently develops.

The results of the inhibition assay showed that in BR patients the antibody response to the enolase peptides CEP‐1 and REP‐1 not only is cross‐reactive and lacking citrulline specificity but also may be of low avidity, thereby explaining the incomplete absorption of anti–REP‐1 activity by the REP‐1 peptide. Although we did not examine antibody avidity in this study, a longitudinal study of patients with presymptomatic RA showed that antibody avidity gradually increased as the onset of joint disease approached 21. Therefore, if certain bacterial infections lead to the initiation of an ACPA response, our study and others 9, 10, 20, 21 suggest that infections such as periodontitis, BR, and others yet to be defined induce a low‐titer, low‐avidity, non–citrulline‐specific ACPA response in the early phases of tolerance breakdown, which subsequently evolves into the higher‐avidity, higher‐titer, and highly citrulline‐specific responses that characterize RA.

A limitation of this study is that the different patient groups were selected from different regions of the UK and were not matched for age. The large number of BR/RA patients in our study does not reflect the prevalence of symptomatic BR in a random population of RA patients, which is probably ∼3% 11, 12. The patients with RA alone were also selected for this study based on the absence of lung disease, and this bias may well reflect the low prevalence of ACPAs in the RA group. However, these results are entirely consistent with a meta‐analysis of several studies 22 and a recent large study, which confirmed a strikingly low frequency (55%) of ACPAs in 230 RA patients without lung disease 23.

In contrast, the more heightened reactivity to citrullinated antigens in patients with BR/RA could represent a certain type of disease in which reactivity to these citrullinated antigens is dominant and perhaps related to the airway disease. Demoruelle et al observed that 76% of anti‐CCP–positive subjects without arthritis had airway abnormalities, including BR, and that articular RA developed in 2 of these subjects within a year 24, and Fischer et al reported that 63% of patients with lung disease without RA had “moderate to high” anti‐CCP positivity 25.

Even though the current study cannot be regarded as an epidemiologic study, we have demonstrated in this substantial population of well‐documented patients that BR could be an unusual but potent model for the induction of autoimmunity in RA by bacterial infection in the lung. To prove that this is a true causal relationship, longitudinal followup of patients with BR is required, and such studies are under way within the UK Clinical Research Network Study Portfolio.

## AUTHOR CONTRIBUTIONS

All authors were involved in drafting the article or revising it critically for important intellectual content, and all authors approved the final version to be published. Dr. Venables had full access to all of the data in the study and takes responsibility for the integrity of the data and the accuracy of the data analysis.

**Study conception and design.** Perry, Kelly, De Soyza, Eggleton, Hutchinson, Venables.

**Acquisition of data.** Quirke, Perry, Cartwright, Kelly, De Soyza, Eggleton, Hutchinson.

**Analysis and interpretation of data.** Quirke, Perry, De Soyza, Eggleton, Hutchinson, Venables.
